# Chikungunya virus replicates in the human testis *ex vivo* and impacts peritubular myoid cells functional markers

**DOI:** 10.1080/22221751.2025.2587984

**Published:** 2025-12-05

**Authors:** Matéo Cartron, Vincent Ciesielski, Laurent Houzet, Krishani Dinali Perera, Hervé Abiven, María José Lista Brotos, Ingrid Plotton, Romain Mathieu, Julien Branchereau, Laurent Martin-Lefevre, Pierre Roques, Dominique Mahé, Nathalie Dejucq-Rainsford

**Affiliations:** a Institut National de la Santé et de la Recherche Médicale, Ecole des Hautes Etudes en Santé Publique, Institut de recherche en santé, environnement et travail, Université de Rennes – UMR_S1085, Rennes, France; b Service de Médecine de la Reproduction, Hôpital Femme Mère Enfant, HCL, Bron, France; c Univ Lyon, Université Lyon 1, INSERM, Stem Cell and Brain Research Institute U1208, Bron, France; d Service d'Urologie, Centre Hospitalier Universitaire de Rennes, Rennes, France; e Service d'Urologie, Centre Hospitalier Universitaire de Nantes, Nantes, France; f Service de coordination des prélèvements, Centre Hospitalier Universitaire de Nantes, Nantes, France; g Center for Immunology of Viral, Auto-Immune, Hematological and Viral Diseases (IMVA-HB/IDMIT), UMR-1184, Université Paris-Saclay, Inserm, CEA, Fontenay aux Roses, France; h Virology Unit, Institut Pasteur de Guinée (IPGui), Conakry, Guinea

**Keywords:** CHIKV, human testis, *ex vivo* model, testis functions, sexual transmission

## Abstract

Chikungunya virus (CHIKV) is an emerging, mosquito-borne alphavirus responsible for debilitating, long-lasting arthralgia and myalgia. In light of recent findings of prolonged CHIKV RNA shedding in human semen and testicular tropism in animals infected with related alphaviruses, it is imperative to investigate CHIKV's capacity to infect the human testis, an established reservoir for arboviruses like Zika, and to delineate its implications for testicular function. Using an *ex vivo* human testicular tissue model, we demonstrate that CHIKV rapidly infects peritubular myoid cells (PMCs) and a range of interstitial cells, with robust viral production peaking at day 3 before declining. Importantly, seminiferous tubule cells and isolated testicular germ cells proved nonpermissive to CHIKV infection, indicating a potential limitation for seminal shedding of virions. Infected testicular explants exhibited a broad antiviral response but limited pro-inflammatory cytokines upregulation. CHIKV replication in the testis induced apoptosis and cell death, with a marked impact on PMC markers including decreased transcriptional expression of genes crucial for PMC contractile properties and extracellular matrix production. In summary, our study highlights the susceptibility of human testicular tissue to CHIKV infection, marked by robust viral replication that primarily compromises PMC function. The observed cellular impairment and damage suggest that CHIKV infection might negatively affect key testicular functions, such as tubular contractility and sperm release. These findings warrant further investigation into semen parameters and viral shedding in CHIKV-infected men.

## Introduction

Chikungunya virus (CHIKV) is a member of the *Togaviridae* family, genus *Alphavirus*, primarily transmitted by *Aedes* mosquitoes. Since 2005, CHIKV has re-emerged beyond its traditional regions in sub-Saharan Africa and Southeast Asia, leading to numerous global outbreaks [1], with over 600,000 reported cases worldwide in 2024 according to the European Centre for Disease Prevention and Control. In 2025, CHIKV is still actively circulating, including an ongoing epidemic on La Réunion Island with an estimated 195,000 cases as of 4th June, 2025 from Santé Publique France records. CHIKV acute infection typically results in viremia for 5–7 days and is symptomatic in 72-95% of cases [2], causing the characteristic chikungunya fever (CHIKF). This illness is marked by high fever, rash, headache, and the hallmark symptoms of myalgia and polyarthralgia [1]. Debilitating musculoskeletal pain can persist in 40-80% of patients for months or even years after the acute phase, significantly impairing their quality of life. Consequently, CHIKV epidemics generate substantial burden on public health and economy. Infected individuals develop a high viremia that can facilitate non-vectorial transmission, e.g. through blood transfusion or contact with other contaminated fluids [3,4]. Following the spread of CHIKV from skin to the bloodstream, animal models evidenced infection of the liver, spleen, joints, and muscles, where viral persistence may contribute to long-lasting muscular and articular disease [5,6]. In fatal human cases, CHIKV RNA and protein were detected in a range of fluids and tissues including cerebrospinal fluid, liver, spleen, kidney, heart and brain [7,8], highlighting CHIKV's broad tissue tropism. There are currently no antivirals approved against CHIKV. Two vaccines have recently received regulatory approval, but their real-world effectiveness, long-term protection and safety across diverse populations remain to be fully established [9]. Recently, CHIKV RNA was detected in the semen of infected men up to 56 days post symptom onset, for some in the absence of detectable viremia, suggesting infection of the male genital tract [10,11]. This observation mirrors previous findings during Zika virus (ZIKV) infections – an arbovirus member of the *Flaviviridae* family, genus *Orthoflavivirus* – where prolonged viral shedding in semen associated with testicular infection were unexpectedly reported [12,13], leading to sexual transmission and transiently altered semen parameters [14–16]. Notably, several other alphaviruses have been identified in the male genital tract of animals [17,18], some sexually transmitted [19]. Therefore, determining whether CHIKV infects the male genital tract is crucial for patient management and evidence-based public health strategies during current and future outbreaks.

The human testis is an immune-privilege organ with two primary functions: the synthesis of sex hormones (steroidogenesis) and the production of spermatozoa (spermatogenesis). Histologically, the organ is divided into two distinct compartments: (i) the interstitial tissue, which contains essentially testosterone-producing Leydig cells, immune cells (mostly M2-like macrophages), fibroblasts and blood vessels and (ii) the seminiferous tubules, surrounded by the contractile myoid peritubular cells, which contain Sertoli cell-nursed germ cells that differentiate into spermatozoa. The immuneprivileged state of the testis, designed to protect developing germ cells from the acquired immune system, is maintained by the blood-testis barrier – a physical barrier of tight junctions between Sertoli cells – and an active local immunosuppressive environment. This unique immunity may contribute to viral evasion from systemic clearance [16].

Here, we aimed to evaluate the capacity of CHIKV to infect the human testis and assess the potential consequences of this infection on sexual transmission and male reproductive functions. To this end, we investigated CHIKV replication in the testis and its effect on testicular cells in a well-characterized *ex vivo* model, previously validated for its relevance to *in vivo* infection by a range of viruses including HIV, Zika virus, SARS-CoV-2 and Mumps virus [13,20–22].

## Materials and methods

### Cell line and viruses

The Indian Ocean Lineage (IOL) CHIKV strain LR2006_OPY1 (GenBank accession No. DQ443544) was isolated during the 2005–2006 outbreak on Reunion Island from a French patient presenting characteristic clinical manifestations of CHIKV infection, including fever, arthralgia, and myalgia [5]. This lineage has been responsible for numerous epidemics across the globe since 2005, including a 2018 outbreak in Thailand [1,23]. The strain was previously passaged 3 times in VeroE6 cells (African green monkey kidney epithelial cells) [5] and we performed an additional single round of amplification to produce viral stocks. Mycoplasma-free VeroE6 cells were infected at an MOI of 0.01 in serum-free medium for 2 h, before addition of complete medium to reach a final serum concentration of 5%. After 4 days of infection, when cytopathic effect was evident, supernatant was centrifuged, filtered (0.45 μm), aliquoted, and frozen at – 80°C.

### Organotypic culture of human testis explants and infection

Testes from post-mortem donors (median age 56 years old, range 29–79 years old) displaying full spermatogenesis as assessed by transillumination were dissected into 3-mm^3^ explants and transferred onto 24-well plates (4 explants/well) containing 500 μL medium (DMEM F12 supplemented with 1× nonessential amino acids, 1× ITS (human insulin, human transferrin, and sodium selenite), 100 U/mL penicillin, 100 μg/mL streptomycin and 10% Fetal Calf Serum (FCS)). All reagents are from Gibco, except FCS from Eurobio

Scientific. Four explants per well were infected in presence of 10^5^ TCID_50_ CHIKV corresponding to 5 × 10^7^ RNA copies of the viral stock, or left uninfected (mock) in 500 µL of culture medium. For each experimental condition, two to three wells were tested. After 4 h incubation, explants were washed 3 times with PBS and transferred onto a polyethylene terephthalate insert (3 μm high-density pores) in 12-well plates (two explants per well) containing 1 mL medium. Three hours later, the medium was changed again to further remove potential residual virus input (time 0 for sample collection). The culture was maintained up to 9 days post-infection in a humidified atmosphere containing 5% CO_2_ at 37°C, with medium collected and fully changed at every collection time point (day 1 (d1), day 2 (d2), day 3 (d3), day 6 (d6) and day 9 (d9)). Media were stored as is or separately in lactate dehydrogenase (LDH) assay buffer at – 80°C. Tissue fragments were fixed in 4% paraformaldehyde or frozen at – 80°C.

### Isolation and infection of testicular peritubular myoid cells and germ cells

Testis fragments were incubated in digesting medium (0.5 mg/ml collagenase I, DNase I 75 μg/ml and SBTI 1 µg/mL, all from Sigma-Aldrich in PBS) for 60 min at 37°C under agitation (110 rpm) to dissociate interstitial tissue from seminiferous tubules. After filtration (100 µm), seminiferous tubules were digested by trypsin (0.25%, 5 ml/g of tissue, 20 min at 37°C under agitation). Trypsin was inactivated, and seminiferous tubules cells were filtered (60 μm) and cultured overnight in culture medium described above for explants. After overnight culture, non-adherent germ cells were removed and cultured in supplemented StemPro as described [24,25]. Adherent cells were cultured for 1–2 weeks in the culture medium described for explants allowing peritubular myoid cells (PMC) to grow. PMC purity was assessed by immunofluorescence detection of PMC marker α-SMA and markers of potential cell contaminants CD68+/CD163 + macrophages, CD34 + cells, FSHR + Sertoli and CYP11A1+ Leydig cells, reaching a PMC enrichment > 80% as determined by cell counting (Figure S1A and B). For CHIKV infection, peritubular cells and germ cells were exposed to the viral inoculum diluted in serum-free medium at a MOI of 1 for 2 h at 37°C. Upon trypsin washes to eliminate residual virus particles, cells were cultured for the periods of time specified in the text and figure legends. For germ cells, non-adherent germ cells were centrifuged and transferred to new medium at 24h post-infection (pi). Culture supernatants were collected and stored at – 80°C. Harvested cells were put onto polylysine-coated glass coverslips and fixed in 4% paraformaldehyde for 20 min at room temperature.

### Testis tissue from CHIKV macaque

We obtained from a previous unrelated study [5] a single PFA-fixed testis from one adult Cynomolgus macaque (*Macaca fascicularis*) inoculated with 10^7^ PFU of the CHIKV LR2006_OPY1 strain by intravenous route. Inoculation of this strain into immunocompetent cynomolgus macaques recapitulated the clinical features of human disease [5]. The animal was euthanized at day 2pi and a range of tissues collected. At that time, the animal blood viral load was > 10^8^ RNA copies/ml and CHIKV antigen detected in the spleen, lymph node and liver [5].

### Determination of viral titer

Viral particles from viral stocks and testis explant or cells supernatants were titrated as TCID_50_/ml on VeroE6 cells based on the method of Spearman and Kärber [26]. Briefly, serial dilutions (100 µl) of cell culture supernatant were added on cells seeded at 1.5 × 10^4^/well in 96-well plates. TCID_50_/mL was calculated by determining the last dilution giving 50% of wells with cells displaying a CPE at day 4.

### RT-qPCR

Total RNA was extracted using QIAamp vRNA (for supernatants) or RNeasy isolation kit (for tissue/cells) and treated with DNase (all from QIAGEN). RNA from tissue/cells was further precipitated with sodium acetate and Glycoblue (both from Invitrogen). RNA extracted from culture supernatants was subjected to RT-qPCR using the GoTaq Probe 1-Step RT-qPCR System (Promega). Primers and probes for CHIKV E1 were used: CHIKV primer forward AAGCTCCGCGTCCTTTACCAAG, CHIKV primer reverse CCAAATTGTCCTGGTCTTCCT, CHIKV probe CCAATGTCTTCAGCCTGGACACCTTT [27]. Viral genome RNA of a known number of copies of vRNA was used as standard curve [5]. Total RNA was reverse transcribed using the iScript cDNA Synthesis Kit (Bio-Rad). Primers for the relative quantification of cellular transcripts are listed in Table S1 and were either used previously [21] or designed using the Primer-BLAST tool. RT-qPCR reactions were performed on a BioRad CFX384 instrument using iTaq SYBR green mix (Bio-Rad) as previously described [21]. Results were calculated by the ΔΔCt method as *n*-fold differences in target gene expression normalized to the reference gene (*ACTB*) and to the mock sample at each time.

### Histology, RNAscope *in situ* hybridization (ISH), and immunohistochemistry (IHC)

Tissues were fixed in 4% paraformaldehyde and embedded in paraffin and either stained with hematoxylin-eosin, labelled with specific antibodies for IHC, processed for RNAScope ISH or dual fluorescence RNAScope-IHC as previously described [13,21,24]. Probes, antibodies and specific conditions are specified in Table S2. Dual staining with α-SMA and CD34 antibodies allowed the discrimination of CD34 + telocytes from CD34 + endothelial cells surrounded by α-SMA + pericytes. As CYP11A1 and cleaved caspase-3 staining required signal amplification in IHC, tissue sections were processed in a Roche automated staining platform (Discovery Ultra, Roche). Conditions used for antibodies against CYP11A1, cleaved caspase-3 and CHIKV capsid are detailed in Table S2. Tyramide signal amplification was applied using Rhodamine (Roche) before labelling with Cy5 (Roche) or FAM (Roche). Further staining with α-SMA, CD34 and/or CD68/CD163 antibodies was done, as detailed in Table S2. Counterstaining was performed using a solution containing DAPI (Southern Biotech). Sections of mock-infected testis explants were systematically used as negative control for CHIKV CA or dsRNA antibodies, and sections stained with isotype antibody control were systematically used as negative control for cell markers or cleaved caspase-3 antibodies. For each mock and infected explants, 3–6 whole tissue explant sections were stained at each time point for each donor.

Colorimetric stained and fluorescent immunostained sections were captured with a scanner NanoZoomer 2.0 RS (Hamamatsu Photonics, Tokyo, Japan at Plateforme H2P2, Biosit) at 20× magnification. Fluorescent images were acquired with the Zeiss Axio Imager system connected to Zen software or with the SP8 confocal system (Leica) connected to LAS software and analyzed using Fiji software.

### Cell counting

For cell quantification in tissue, 3–6 whole tissue explant sections per donor and time point were analyzed. Scanned images from NanoZoomer slide scanner were imported and processed using QuPath-0.5.0 tissue quantification open source software [28].

For automated counting, illustrated in Figure S2, nuclei segmentation was performed based on DAPI staining using a Stardist model [29] trained with manually annotated nuclei [30–32]. Input parameters (pixel size = 0.6 µm, minimum area = 3µm^2^, maximum area = 120µm^2^ and probability threshold = 0.09) were adjusted for optimal detection of the different cell type in testicular tissue. A cytoplasm region for each cell was obtained as a dilation of the nucleus area to measure cytoplasmic fluorescence marker levels for cell classification. For CHIKV CA + α-SMA + PMCs and CHIKV CA + CD34 + telocytes, blood vessels were manually excluded for each whole section thanks to α-SMA + and CD34 + staining, defining the regions to be analyzed. A subset of small representative regions of immunostained sections from mock and infected explants at different time points were selected for each donor to train a classifier for identifying positive cells for fluorescent markers. Each classifier was finally applied to segmented cells, allowing the precise quantification of single positive cells (CHIKV CA+, α-SMA + PMCs and CD34 + telocytes) and double positive cells (CHIKV CA + α-SMA + PMCs or CHIKV CA + CD34 + telocytes) (Figure S2). Automated counting was performed on both mock and CHIKV-infected testis explants.

For CHIKV CA + CYP11A1+ Leydig cells and CHIKV CA + CD68/CD163 + macrophages were manually counted in QuPath software on at least 3 whole tissue sections, as their very low number did not enable to train a cell classifier. Cleaved caspase-3 positive cells were also manually counted in interstitial tissue of explants.

### Viability assay

Global tissue viability was assessed by measuring the activity of released lactate dehydrogenase (LDH) in culture medium using the enzymatic LDH-Glo™ Cytotoxicity Assay (Promega) according to manufacturer’s protocol.

### Immunocytofluorescence

Primary testicular cells (peritubular or testicular germ cells) were put onto polylysinecoated glass coverslips and fixed in 4% paraformaldehyde for 20 min at room temperature. Immunocytofluorescence was performed as previously described [13,21]. Conditions used for antibodies are detailed in Table S2. Slides were counterstained with ProLong medium containing DAPI (Invitrogen). Isotype control antibodies or non-infected cells were used as negative controls. Images were acquired with the Zeiss Axio Imager system connected to Zen software and analyzed using Fiji software.

### Hormone release measurement

Testosterone was measured using an in-house liquid chromatography-mass spectrometry after liquid extraction using Agilent’s 1290 Infinity II LC System and MSMS 6495. Mean intra-assay coefficients of variation were 3.6% and 2.8% for testosterone concentrations of 2.15 and 8.26 nmol/L, respectively; inter-assay coefficients of variation were 4.71% and 5.98% for testosterone concentrations of 2.254 and 8.934 nmol/L, respectively. Inhibin B concentration was determined using Beckman Coulter’s Gen II ELISA Kit according to the manufacturer’s instructions. The limit of quantification was 5 ng/L.

### Statistics

The statistical tests used for data analysis are specified in the figure legends. All analyses were conducted using GraphPad Prism 9.5.1, a significance threshold of *p* < 0.05 was applied.

### Study approval

Normal testes were obtained at autopsy of organ donors and processed within 2 h of surgery. The procedure was approved by Ethics

Committee Ouest V, Rennes, France (authorization DC222 2022-5253), and the French National Agency for Bio-medical Research (authorization PFS23-001).

## Results

### Human testis explants support robust CHIKV replication

Testis explants from seven uninfected donors (age range, 29-79) exposed to CHIKV for 4 h were cultured up to 9 days at the air/medium interface, with medium fully changed at every indicated time points. CHIKV RNA released in testis explants supernatants significantly increased between day 1 post-infection (dpi) (median: 1.23 × 10^6^ copies/mL; range 5.59 × 10^5^–4.67 × 10^6^ copies/mL) and d3pi (median: 1.91 × 10^8^ copies/mL; range 7.98 × 10^7^–4.71 × 10^8^ copies/mL), while a tendency for decline was observed between d6pi (median: 1.45 × 10^8^ copies/mL; range 9.42 × 10^7^–1.99 × 10^8^ copies/mL) and d9pi (median: 6.65 × 10^7^ copies/mL; range 5.04 × 10^7^–1.08 × 10^8^ copies/mL) (Figure 1(A)). CHIKV RNA release by explants consistently exceeded input vRNA (5 × 10^7^ copies/mL), with a median cumulative vRNA release reaching 5.01 × 10^8^ copies/mL (range 3.63 × 10^8^- 1.12 × 10^9^ copies/mL). Supporting these findings, *in situ* hybridization for CHIKV genomic RNA in infected testis explants revealed extensive labelling from d2pi up to d9pi, in contrast to sparse detection at d1pi. Throughout the culture, CHIKV RNA was essentially localized within the interstitial tissue, with only a few spots observed in some seminiferous tubules (Figure 1(B)).

**← Figure 1.**
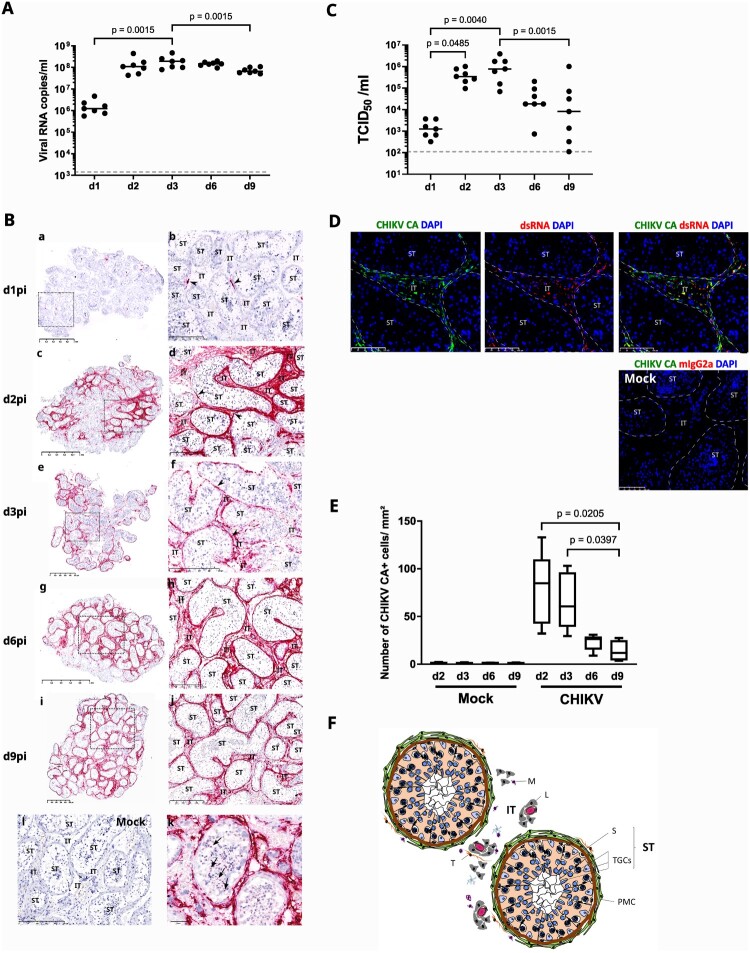
Human testis explants support CHIKV replication within the interstitial tissue. Human testis explants from seven donors were infected with the Chikungunya virus (CHIKV) Indian Ocean Lineage (IOL) strain LR2006_OPY1 at a concentration of 2 × 10^5^TCID_50_/mL for four hours, as described in materials and methods. (A) CHIKV E1 mRNA was quantified by RT-qPCR in culture supernatants. The time points at d1, d2 and d3pi correspond to infectious virions release in culture supernatants over 24 h interval, while d6 and d9pi correspond to cumulative viral release over a 3-day period. Each dot represents a donor and bars represent the medians. Dotted line represents the threshold of detection. All mock explants were negative. Statistical analysis was conducted using the non-parametric Friedman test followed by Dunn’s multiple-comparison test on d1, d2 and d3pi values, or between cumulative amount at d3 vs d6 or d9pi to compare similar release duration. (B) RNAscope *In Situ* Hybridization (ISH) was performed using a probe targeting CHIKV genomic E1 RNA at the indicated time points (a-k). Representative images of whole explant sections, with regions highlighted by dotted-line square (a, c, e, g, i) enlarged on the right (b, d, f, h, j). Few positive spots observed in rare seminiferous tubules are indicated by an arrow (k). Representative image of RNAScope ISH in control mock tissue (l). (C) CHIKV titers were measured in the supernatants of testis explant cultures over time. The time points at d1, d2 and d3pi correspond to infectious virions release in culture supernatants over 24 h interval, while d6 and d9pi reflect cumulative viral release over a 3-day period. Each dot represents an individual donor, with bars indicating median values. Dotted line represents the threshold of detection. All mock-infected explants tested negative. Statistical analysis was conducted using the non-parametric Friedman test followed by Dunn’s multiple-comparison test on d1, d2 and d3pi values, or between cumulative amount at d3 vs d6 or d9pi to compare similar release duration. Each dot represents a donor and bars represent the medians. (D) Immunohistochemistry using a specific antibody against. CHIKV capsid (CHIKV CA) or against replicative RNA (dsRNA) was performed on testis explants. Representative images of immunostained sections at peak viral load on d2pi are shown. No staining was ever observed in mock-infected testis explants. Nuclei are stained in blue. (E) The number of CHIKV CA + infected cells was quantified in at least 3 whole testis sections per condition from four donors at the indicated time points, as described in materials and methods section. Boxplot shows the minimum and maximum values with central lines indicating the median. Statistical analyses were conducted using the non-parametric Kruskal-Wallis test followed by Dunn’s multiple-comparison test to compare each timepoint to one another. (F) Schematic of the testis histology, with its two compartments: (i) the interstitial tissue (IT), which contains primarily testosterone-producing Leydig cells (L), macrophages (M), telocytes (T), and blood vessels; (ii) the seminiferous tubules (ST), where testicular germ cells (TGCs) at different stages of differentiation are embedded within Sertoli cells and surrounded by contractile peritubular myoid cells (PMCs).

In agreement with these data, infectious viral particles quantified by TCID_50_ assay in culture supernatants increased significantly from d1pi (median: 1.26 × 10^3^ TCID_50_/mL; range 3.27 × 10^2^–3.68 × 10^3^ TCID_50_/mL) to d2pi (median: 3.46 × 10^5^ TCID_50_/mL; range 9.53 × 10^4^–1.01 × 10^6^ TCID_50_/mL) and d3pi (median: 7.73 × 10^5^ TCID_50_/mL; range 6.91 × 10^4^–3.87 × 10^6^ TCID_50_/mL). The viral titer significantly decreased thereafter to reach a median of 8.21 × 10^3^ TCID_50_/mL (range 1.12 × 10^2^–1 × 10^6^ TCID_50_/mL) at d9pi (Figure 1(C)). Evidence for CHIKV replication in the testis was further supported by the *in situ* identification of productively-infected cells through CHIKV capsid (CHIKV CA) staining and replicative double strand (ds) RNA, shown here at d2pi (Figure 1(D)). Both CHIKV antigen and replicative RNA were exclusively detected in the interstitial tissue of the testis throughout culture (Figure 1(D)), suggesting that the detection of CHIKV genomic RNA in rare tubules reflected low levels of nonreplicative viral particles. The number of CHIKV capsid-positive infected cells quantified at d2pi and d3pi significantly declined by d9pi (Figure 1(E)), mirroring the pattern observed for TCID^50^ measurements. We analyzed a fixed testis sample collected from one cynomolgus macaque infected by CHIKV via the intravenous route, as part of a previous unrelated study [5]. CHIKV genomic RNA and capsid antigen were detected *in vivo* within the interstitial tissue and peritubular area of this animal as early as d2pi (Figure S3). This suggests rapid testicular dissemination of CHIKV *in vivo*, exhibiting a tropism consistent with that seen in *ex vivo*-exposed human testis explants.

Taken together, these results demonstrate that CHIKV robustly replicates within the interstitial tissue of the human testis *ex vivo,* reaching a peak of infected cells number and infectious virions production within the first 3 days post-exposure. *In vivo,* CHIKV infected the testis of an experimentally-infected macaque as early as 2 days post-exposure, showing similar exclusive interstitial and peritubular localization.

### Peritubular myoid cells are primary targets of CHIKV in human testis explants, alongside telocytes, resident macrophages, and a few Leydig cells

**Figure 2.**
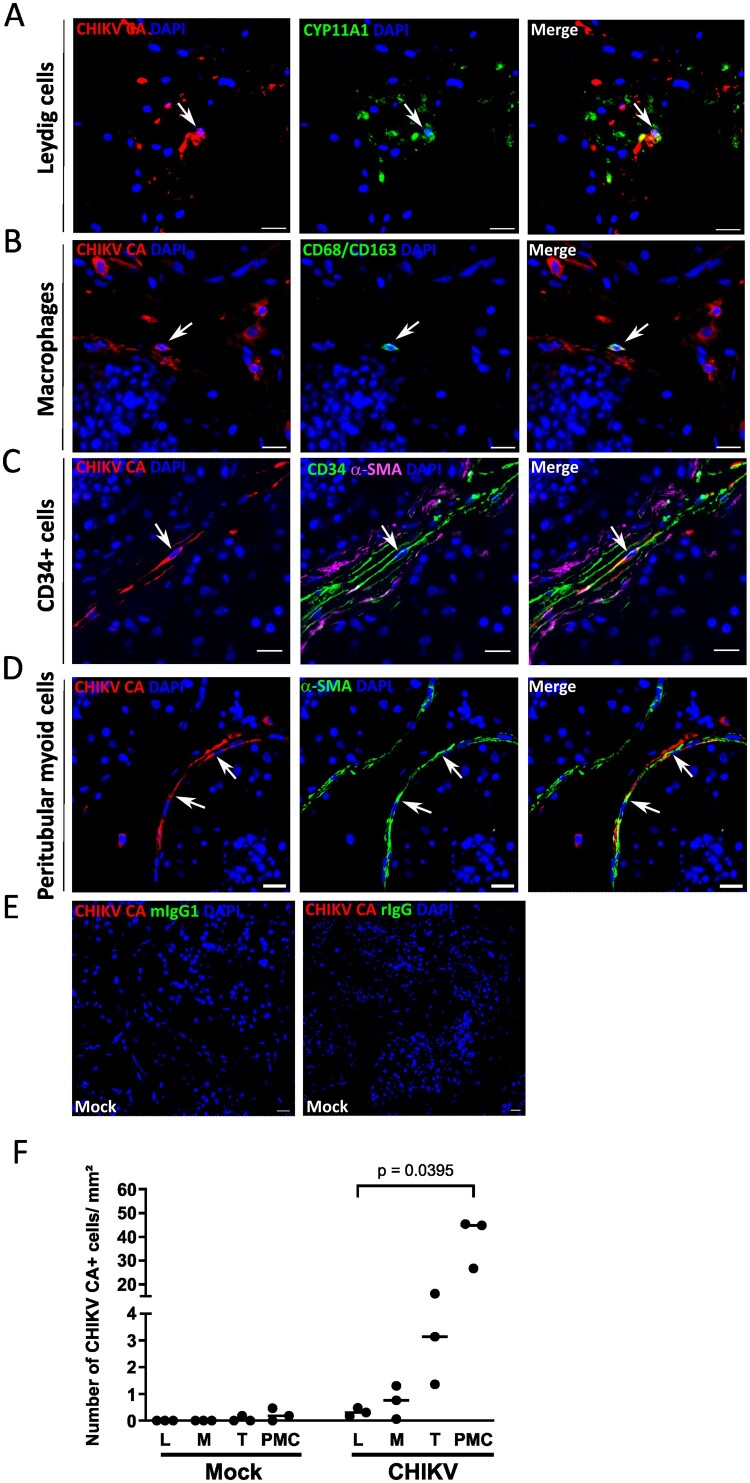
CHIKV infects peritubular myoid cells and a range of interstitial cells in testis explants. The characterization of CHIKV target cells was undertaken at d2pi by combining antibody against CHIKV CA with antibodies against (A) CYP11A1 (Leydig cell marker) (B) CD68/CD163 (macrophage marker), (C) CD34 and α-SMA (interstitial CD34+ α-SMA – telocytes) and (D) α-SMA (peritubular myoid cell marker). (E) No IHC staining for CHIKV, nor signal from cell marker isotypes, was detected in mock-infected testes. Nuclei are counterstained in blue. Scale bars, 20 μm. (F) The number of CHIKV CA + CYP11A1+ Leydig cells (L), CHIKV CA + CD68 + CD163 + macrophages (M), CHIKV + CD34 + telocytes (T) and CHIKV CA + α-SMA – peritubular cells (PMC) was quantified in 3 whole testis sections from three donors at d2pi, as described in materials and methods section. IHC staining using CHIKV CA antibody in mock-infected explants combined with control isotype antibody for cell markers was used as control. Each dot represents an individual donor, with bars indicating median values. Statistical analysis was conducted using the non-parametric Kruskal-Wallis test followed by Dunn’s multiple-comparison test.

We further characterized the nature of CHIKV-infected cells in the testis by combining antibodies against CHIKV capsid, dsRNA and specific cell markers (Figure 2 and Figure S4). CHIKV-capsid antigen (Figure 2) and replicative RNA (Figure S4) were evidenced in few CYP11A1+ Leydig cells (Figure 2(A) and Figure S3A) and CD68/CD163 + macrophages (Figure 2(B) and Figure S3B) but also in CD34 + interstitial telocytes (identified based on CD34+ α-SMA – staining) (Figure 2(C) and Figure S3C), and α-SMA + peritubular myoid cells (PMCs) (Figure 2(D) Figure S3D). The quantification of infected cell types undertook at the peak of infection (d2pi) demonstrated that PMCs bordering the seminiferous tubules were the primary targets of CHIKV within testis explants, followed by telocytes, macrophages and Leydig cells (Figure 2(F)). Notably, no signal was ever detected for either CHIKV capsid or replicative RNA inside the seminiferous tubules. Similarly to human testis explants, CHIKV CA co-localized with α-SMA + peritubular cells in the testis of the 2 d-infected macaque (Figure S4F). While infected PMCs remained detectable until the end of testis explants culture at d9pi (Figure S5A–D), quantification evidenced their significant decrease from d3 to d9pi (Figure S5E). Although non-significant, a similar pattern was observed for the minor targets, telocytes and macrophages (Figure S5E). To investigate the expression of cellular receptors previously described to mediate CHIKV entry, such as MXRA8 and glycosaminoglycans, we analyzed publicly available single-cell RNA sequencing (scRNA-seq) data using the Reprogenomics Viewer [33]. These data indicate strong and broad *MXRA8* expression by peritubular myoid cells and mesenchymal cells, in contrast to weak or lack of *MXRA8* expression by Leydig cells, immune cells, perivascular cells, Sertoli cells and germ cells (Figure S6). Unfortunately, this expression pattern could not be confirmed at the protein level, as despite using two commercial polyclonal antibodies, all our attempts to detect MXRA8 protein in testis and various control organs (prostate, colon) and cell lines (HepG2, U2OS, MCF7) failed. Other CHIKV putative receptors transcripts such as PHB and BSG were widely expressed across testicular cell types, whereas MARCO and TLR4 expression was essentially restricted to testicular immune cells (Figure S6).

We next exposed PMCs isolated from human testes to CHIKV, which confirmed efficient viral replication in these cells, as evidenced by elevated vRNA and TCID^50^ at d1 and d2pi (Figure 3(A, B)), as well as by dsRNA detection in α-SMA + cells in immunofluorescence (Figure 3(C)). Notably, a marked cytopathic effect occurred at d2pi (Figure 3(D)).

**← Figure 3.**
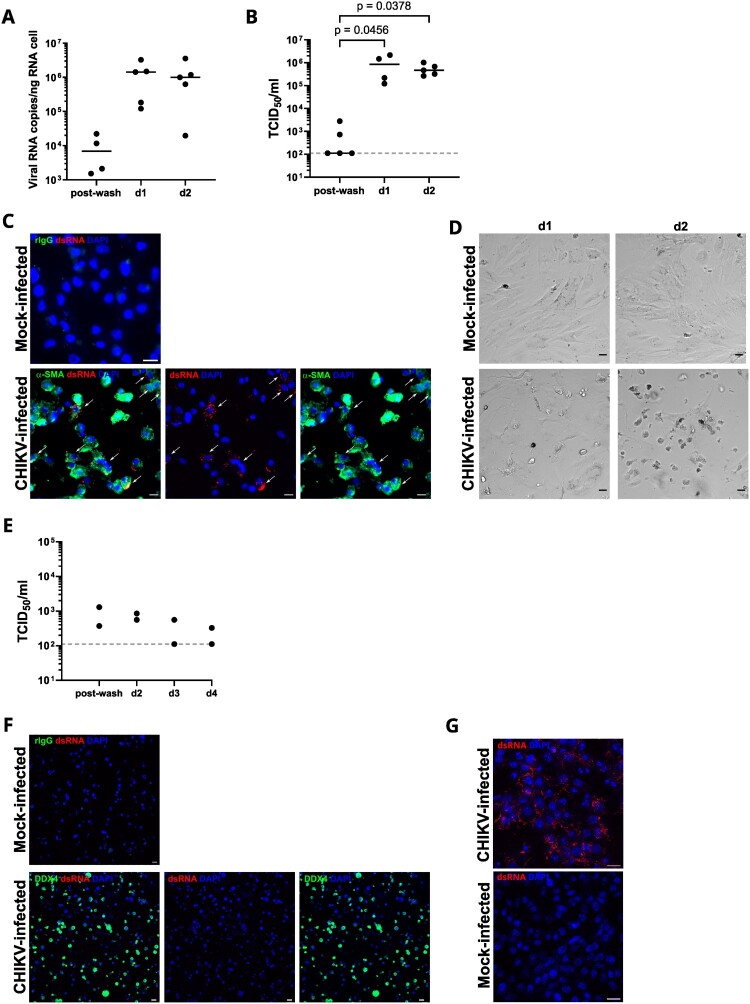
CHIKV replicates in human peritubular myoid cells but does not infect DDX4 + testicular germ cells *in vitro*. (A-D) Primary PMCs and (E-G) testicular germ cells were infected or mock-infected with CHIKV at a MOI of 1 and cultured respectively up to 2 – and 4-days post-infection. (A) CHIKV RNA was quantified by RT-PCR in PMC total RNA. No viral RNA was detected in mock-infected samples. Each dot corresponds to a distinct donor, and horizontal bars indicate median values. (B) CHIKV infectious titers were measured in PMC culture supernatants using an infectivity assay on VeroE6 cells. The time points at d1 and d2pi correspond to cumulative viral release. Each dot represents an individual donor, with bars indicating median values. Dotted line indicates threshold of detection. All mock-infected cells tested negative. Statistical analysis was conducted using the non-parametric Kruskal-Wallis test followed by Dunn’s multiple-comparison test. (C) Immunocytofluorescence of CHIKV or mock-infected PMCs using antibodies targeting α-SMA and replicative RNA (dsRNA) at d1pi. Nuclei are stained in blue. Results are representative of two different donors. Scale bars, 20 μm. (D) Brightfield microscopy of CHIKV or mock-infected PMCs at d1 or d2pi, representative of 4 different donors. (E) CHIKV infectious titers were measured in testicular germ cells culture supernatants using an infectivity assay on VeroE6 cells. Dots represent individual donors (n = 2). Dotted line indicates threshold of detection. All mock-infected cells tested negative. (F) Immunofluorescence of CHIKV-infected testicular germ cells stained using DDX4 and replicative RNA (dsRNA) antibodies at d3pi. Mock-infected testicular germ cells stained with anti-dsRNA antibody and IgG isotype are shown as a negative control. Nuclei are stained in blue. Scale bars, 20 μm. (G) Immunofluorescence of CHIKV-infected VeroE6 cells stained with replicative RNA (dsRNA) antibodies were used as a positive control. No staining was observed in mock-infected Vero cells. Nuclei are stained in blue. Scale bars, 20 μm.

To investigate whether the absence of CHIKV detection within the seminiferous tubules was due to limited virus diffusion or to the intrinsic non-permissiveness of tubular cells, we infected freshly isolated testicular germ cells from two donors and cultured them up to day 4. The lack of productive infection of germ cells was evidenced by the absence of infectious particles release increase in the supernatant of purified testicular germ cells exposed to CHIKV (Figure 3(E)). Immunofluorescence using antibodies against dsRNA and markers for germ cells (DDX4) concordantly showed no evidence of CHIKV replication in testicular germ cells at d3pi (Figure 3(F)), in contrast to permissive VeroE6 cells (Figure 3(G)).

Altogether, these results demonstrate that CHIKV infects a range of testicular interstitial cells, primarily targeting peritubular myoid cells, whereas testicular germ cells (an established reservoir for ZIKV [12,13]) did not support CHIKV replication *ex vivo* and *in vitro*.

### CHIKV infection elicits a dominant antiviral response and subdued pro-inflammatory cytokine upregulation in human testis explants

**Figure 4.**
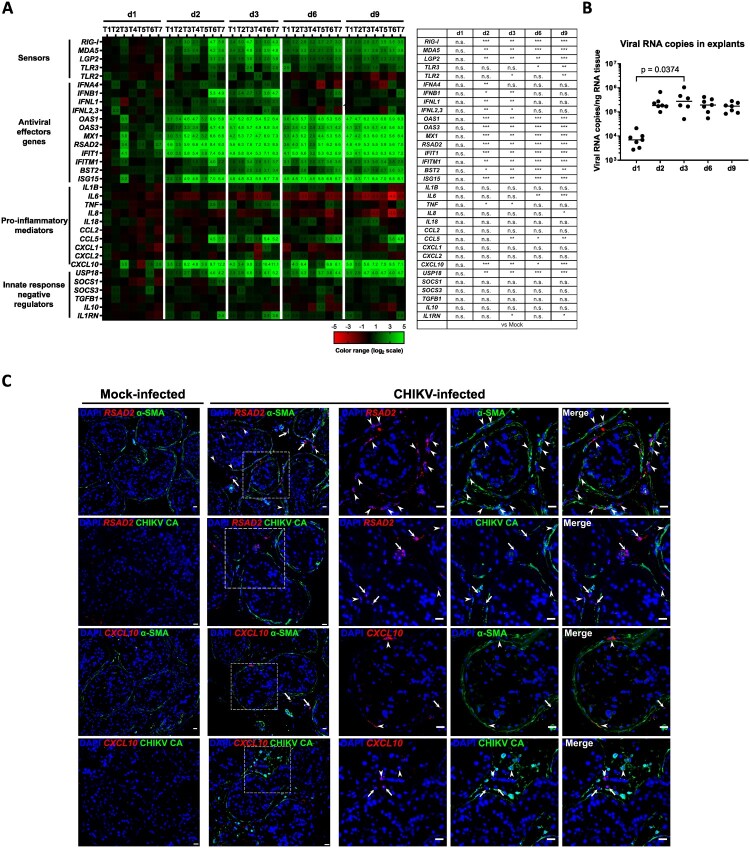
CHIKV infection induces a broad range of antiviral effectors but a limited pro-inflammatory response in testis explants. (A) Innate immune gene expression was assessed by RT-qPCR in testis explants from six to seven donors infected with CHIKV for the indicated durations. The heatmap displays log2-transformed expression ratios comparing CHIKV-infected samples to time-matched mock-infected controls, with green indicating upregulation and red representing downregulation relative to controls. Statistical analyses were conducted using the non-parametric Mann-Whitney test and presented as a table. (B) Viral loads in testis explants analyzed in (A) were quantified by RT-qPCR targeting E1 mRNA. No viral RNA was detected in mock-infected samples. Each dot corresponds to a distinct donor, and horizontal bars indicate median values. Statistical significance was determined using the non-parametric Kruskal-Wallis test followed by Dunn’s multiple-comparison test. (C) RNAscope ISH was performed using a probe targeting either RSAD2 or CXCL10 RNA combined with immunohistochemistry using α-SMA or CHIKV CA antibodies, in CHIKV or mock-infected explants at d2pi (*RSAD2*) or d3pi (*CXCL10*). Arrows show single *RSAD2* + or *CXCL10* + cells in the interstitial tissue, while arrowheads indicate *RSAD2* + cells co-labelled with α-SMA + or CHIKV CA+, and *CXCL10* + cells colabelled with α-SMA + or CHIKV CA + . Enlarged regions highlighted by dotted-line square are shown on the right. Scale bars, 20 μm.

To analyze the innate immune response of the human testis to CHIKV infection, we measured the expression of a panel of pathogen sensors and innate immune effectors by RT-qPCR in CHIKV – versus mock-infected testis explants. Transcripts encoding a broad range of antiviral effector genes (among which *OAS3*, *RSAD2, IFIT1*, *IFITM1*, *BST2* and *ISG15*, all known to interfere with CHIKV replication [34]), as well as viral RNA sensors *RIG-I*, *MDA5* and *LGP2*, were significantly upregulated in CHIKV-infected testis explants from d2pi onwards (Figure 4(A)), concomitantly to the increase in viral RNA levels (Figure 4(B)). Expression of *IFNB1*, a key type I interferon for the amplification of the antiviral response, was also elevated at d2pi and/or d3pi depending on donors (Figure 4(A)). The type I IFN negative regulator *USP18* was significantly upregulated from d2pi onward. *CXCL10* was the only proinflammatory cytokine significantly upregulated from d2 to d9pi, while regulators of inflammation (*SOCS1*, *SOCS3*, *TGFB1*, *IL10)* were not or only minimally induced. *In situ,* the transcripts encoding *RSAD2,* the most induced antiviral effector (Figure 4(A)), and the pro-inflammatory chemokine *CXCL10* localized within α-SMA + PMCs and interstitial cells and were evidenced in both infected and bystander cells (Figure 4(D)).

**Figure 5.**
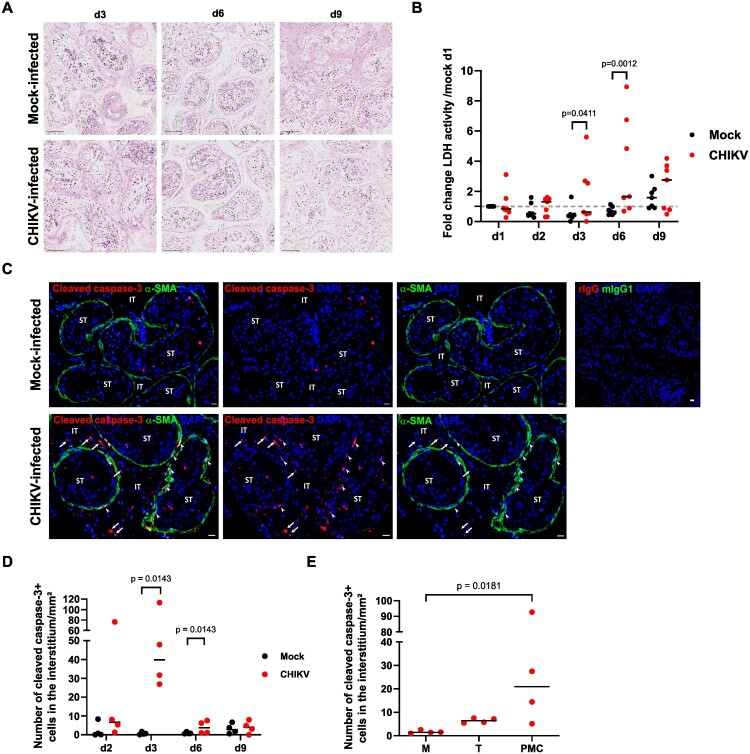
CHIKV infection induces apoptosis in peritubular myoid cells and interstitial cells. (A) Hematoxylin-eosin histological staining representative of three donors was performed from d3 to d9pi in CHIKV – and mock-infected explants. Scale bars, 100 µm. (B) LDH activity measured in supernatants from CHIKV – and mock-infected testis explants during culture was expressed as a fold change of mock-infected explants at d1 of culture. Each dot represents a different donor. Testis explants from six to seven donors were analyzed, with at least 3 replicates per condition for each donor. Non parametric Mann-Whitney test was used to compare LDH activity. (C) Immunohistochemistry was performed using antibody against cleaved caspase-3 combined with α-SMA antibody in CHIKV – or mock-infected explants at d3pi. Arrows show cleaved caspase-3 + cells in the interstitial tissue, while arrowheads indicate double positive α-SMA+/ cleaved caspase-3 + peritubular myoid cells. No IHC staining with control isotype antibodies was detected in mock-infected testes. Scale bars, 20 μm. (D) Immunostained cleaved caspase-3 positive cells in the interstitial tissue of CHIKV or mock-infected explants across culture. Non parametric Mann-Whitney test was used to compare the number of cleaved caspase-3 positive cells in CHIKV versus mock-infected explants. (E) Immunostained cleaved caspase-3 + cells colabelled with either CD68 + CD163 + macrophages (M), CD34 + telocytes (T) or α-SMA + peritubular myoid cells (PMC) in the interstitial tissue of CHIKV at d3 from the same 4 donors were quantified as described in materials and methods section. Non-parametric Kruskal-Wallis test followed by Dunn’s multiple-comparison test was performed to compare the number of cleaved caspase-3 positive between cell types. Each dot represents an individual donor, with bars indicating median values.

**Figure 6.**
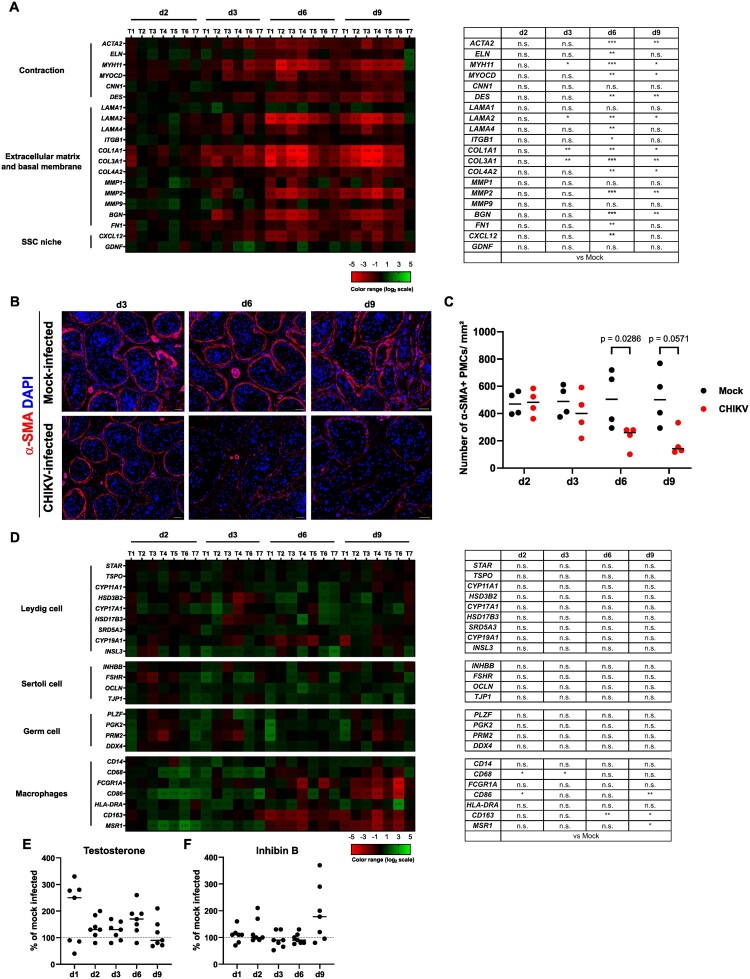
CHIKV infection alters peritubular myoid cells viability and key functional markers. (A) mRNA expression profiles of PMC markers involved in their contractile properties, production of extracellular matrix and basal membrane components and interaction with the spermatogonial stem cell niche (SSC niche markers) were quantified by RT-qPCR in CHIKV and mock-infected testis explants from d2 to d9pi. Values were normalized on actin reference gene (*ACTB)* and expressed as fold changes of mock-infected explants. The heatmap displays log2-transformed expression ratios comparing. CHIKV-infected samples to time-matched mock-infected controls, as described in Figure 4(A). Statistical analyses were conducted using the non-parametric Mann-Whitney test and presented as a table. (B) Immunohistochemistry using anti – α-SMA antibody was performed on CHIKV and mock-infected testis explants from d3 to d9pi. Nuclei are stained in blue. (C) Quantification of immunostained α-SMA + peritubular myoid cells in CHIKV or mock-infected explants from 4 donors from d2 to d9pi was performed as described in material and methods section. Each dot represents a different donor, horizontal bars represent the median values. (D) mRNA expression profiles of markers for Leydig cells, Sertoli cells, testicular germ cells and macrophages quantified by RT-qPCR from d2 to d9pi in CHIKV versus mock-infected testis explants and represented as in (A). (E-F) Testosterone (E) and inhibin B (F) were measured in CHIKV and mock-infected testis supernatants from 7 donors and expressed as percentage of mock-infected explants on the corresponding day of culture. Each dot represents a different donor, horizontal bars represent the median values. Statistical analyses were conducted using the non-parametric Mann-Whitney test.

These data indicate that CHIKV infection triggers a broad antiviral innate response from d2pi in testis explants, which may help contain the infection, whereas the pro-inflammatory response is limited. CHIKV infection alters cell viability and peritubular myoid cell markers in human testis explants The impact on testicular morphology and cell viability of CHIKV *ex vivo* infection was next assessed. While the overall tissue integrity was maintained over the 9-day culture in CHIKV – and mock-exposed explants (Figure 5(A)), a significant increase of lactate dehydrogenase (LDH) activity in supernatants from mock – versus CHIKV-infected testis explants was observed at d3 and d6pi, revealing cell damages (Figure 5(B)). The *in situ* detection of cleaved caspase-3 (Figure 5(C)) revealed an elevated number of apoptotic interstitial cells at d3pi in CHIKV infected explants, of which a high number of α-SMA + PMCs compared to macrophages or telocytes (Figure 5(D, E)). As expected, only a few apoptotic cells were observed in the seminiferous tubules of both mock – and CHIKV-infected explants. The transcriptional expression of several functional markers of PMCs was significantly decreased (from d3 to d9pi depending on the markers), including genes involved in PMC contraction such as *ACTA2*, *ELN*, *MYH11*, *MYOCD*, *DES* (Figure 6(A)), as well as a range of basal membrane and extracellular matrix components such as collagen I (*COL1A1)*, III *(COL3A1*) and IV (*COL4A2*), metalloprotease (*MMP2*) and glycoproteins interacting with collagen (*BGN*), fibronectin (*FN1*). Notably, except for the laminin subunit α2 *LAMA2* and integrin β1 *ITGB1*, the expression of basal membrane factors also produced by Sertoli cells or germ cells (e.g. *LAMA1*, *MMP1* and *MMP9*) [35,36] were not modified. Among key genes involved in the maintenance of the spermatogonia stem cell niche (SSC) by PMCs [37], *CXCL12* was significantly decreased at d6pi, whereas *GDNF* expression was unchanged. At the protein level, we observed diminished α-SMA staining intensity in infected versus mock-infected testis explants from d6pi onwards (Figure 6(B)), along with significantly reduced numbers of α-SMA + PMCs (Figure 6(C)), in agreement with the diminished expression of α-SMA's encoding gene, *ACTA2* (Figure 6(A)).

The effect of CHIKV infection on the other testicular cell types was examined through the measurement of cell markers transcriptional expression and testicular hormones release. In line with the lack of impact of CHIKV infection on a range of Leydig cell markers including steroidogenic enzymes (Figure 6(D)), testosterone levels in CHIKV-infected explants did not significantly differ from those of mock-infected explants throughout the culture (although a trend for a transient increase was observed at d1pi) (Figure 6(E)). Furthermore, CHIKV infection did not alter the transcript levels of Sertoli cell markers (Figure 6(D)) nor the release of inhibin B (Figure 6(F)). Germ cell markers were also unmodified by the infection during the culture time frame (Figure 6(D)). While a subset of macrophage markers, specifically *CD86* at d9pi, *CD163* at d6 and d9pi, and *MSR1* at d9pi, showed decreased expression, others (*CD14*, *FCGR1A* and *HLA-DRA*) remained unaffected (Figure 6(D)). We even detected an upregulation of some of their markers including *CD68* at d2 and d3pi as well as *CD86* at d2pi.

Taken together, these results indicate that CHIKV infection elicits apoptosis in testicular cells *ex vivo*, with a notable propensity for PMCs. The impact of CHIKV infection on the expression of PMC-specific functional markers and a subset of macrophage markers indicates a disruption of the testicular homeostasis.

## Discussion

The unexpected sexual transmission of Zika virus (a member of the *Flaviviridae* family) by recovered men has underscored the importance of investigating other arboviruses for potential seminal excretion. Specifically, the detection of chikungunya virus RNA in semen of infected men up to 56 days post-symptom onset, coupled with evidence of testicular tropism and sexual transmission for related alphaviruses in animal models [10,11,18,19], prompted us to explore whether CHIKV (an alphavirus member of the *Togoviridae* family) can infect the human testis – an established reservoir for ZIKV [13].

Here, we demonstrate that CHIKV (IOL strain isolated in 2006) rapidly replicated to high levels in human testis interstitial tissue *ex vivo*, primarily targeting PMCs, resident macrophages and telocytes, with lesser involvement of Leydig cells. PMCs susceptibility to CHIKV infection aligns with their fibroblast and smooth muscle-like properties [38] and is consistent with prior findings identifying fibroblasts and muscle cells as primary CHIKV targets [5,39,40]. While macrophages represent another key cell type targeted by CHIKV, our discovery of CHIKV infecting telocytes in the testis is a new finding, as this cell type has never been associated with CHIKV infection. Telocytes, a recently identified cell type with not fully understood functions [41], have long, network-forming cytoplasmic processes (telopodes) connected to numerous other cell types that could contribute to virions spreading in the testis interstitial tissue. Conversely and most importantly, we found no evidence of replicative RNA or capsid protein within seminiferous tubules. This suggests that although CHIKV genome was detected in a few tubules, the virus fails to replicate in Sertoli cells and germ cells (a cell reservoir for ZIKV, associated with seminal excretion). This conclusion is further supported by the absence of CHIKV replication in isolated human testicular germ cells and poor transcriptional expression of CHIKV receptor MXRA8 in germ cells and Sertoli cells, in contrast to its strong expression in PMCs. The detection of CHIKV RNA and protein within PMCs of a CHIKV-infected macaque, as early as two days post-intravenous inoculation, corroborates the cellular tropism of CHIKV in human testis explants and demonstrates the virus's rapid dissemination to the testis during peak viremia *in vivo*. Experimentally infected macaques recapitulate many of the viral, clinical and pathological features observed in human CHIKV disease [5]. Unfortunately, we only had access to one CHIKV-infected macaque’s testis, precluding any conclusion on the frequency and duration of testis infection *in vivo*. Moreover, the single animal from which testis was positive for CHIKV *in situ* exhibited a high blood viral load above 8 log_10_ RNA copies/ml. Notably, testicular tropism was recently reported for two other emergent zoonotic alphaviruses: Mayaro virus in Rhesus macaques infected for 10 days, and Getah virus in a mouse model, the latter showing interstitial localization from 12hpi up to d14pi, a timing after which testis infection vanished [17,18].

Altogether, these data suggest that testicular involvement could be a common feature among certain alphaviruses.

Notably, the production of infectious CHIKV particles by testis explants, after reaching a peak at d3pi, significantly declined at the end of the culture period. This is in contrast with ZIKV, which steadily replicated at a lower level up to d10pi in testis explants, while using similar viral dose [13]. Unlike with ZIKV also, a decrease in cell viability was observed upon CHIKV infection, with a peak in apoptotic cells at d3pi and an increase in cell death measured by LDH activity between d3 and d6pi. Apoptotic cell death as a consequence of CHIKV infection has been previously reported both *in vitro* [42,43] and *in vivo* [6]. Nevertheless, the number of apoptotic cells in testis explants (median of 39 cleaved caspase3 positive cells/mm²) at d3pi was below the number of infected cells (median of 60 infected cells/mm²), which might be explained by the ability of CHIKV to induce a cytoprotective effect through the manipulation of autophagy, or a delayed effect [44,45]. In addition to cell death, antiviral immunity could play an important role in the *ex vivo* control of CHIKV replication in the testis. Indeed, testis explants responded to CHIKV infection with a broad antiviral signature from d2 onwards, which in addition to *IFNB1* upregulation, included a range of antiviral effectors (eg *ISG15*, *RSAD2*, *OAS3*, *IFIT1*, *IFITM1*, *BST2*) known to limit CHIKV replication *in vitro* and *in vivo* [34,42,46–48]. The detection of *RSAD2* in CHIKV-infected but also bystander cells suggests that IFN-I signalling may contribute to restrict CHIKV infection in the testis explants by protecting uninfected cells. Altogether, our data suggest that CHIKV replication in the testis may be controlled by cell death and/or innate immune responses. However, the 10-day limited lifespan of our *ex vivo* model does not allow for viral persistence analysis. Therefore, although the decline in viral production at d9pi in testis explant suggest that CHIKV replication may not be sustained, the persistence of the virus at low level in subsets of infected testicular cells that resisted death cannot be ruled out. Indeed, both fibroblasts and macrophages have been shown to harbour CHIKV RNA or antigens for extended durations in other tissues [5,39].

The pro-inflammatory response of CHIKV-infected testis explants was minimal, being restricted to the upregulation of *CXCL10*, along with *CCL5* in some donors. This contrasts with the strong proinflammatory response of isolated synovial fibroblasts or fibroblast-like synoviocytes infected by CHIKV [49,50]. Interestingly, we previously reported a similar weak pro-inflammatory response in testis explants infected with ZIKV and SARS-CoV-2 [13,21], whereas mumps virus, which replicated to high levels in testis explants, rapidly induced the release of IL-1β and IL-18 in testis explants [22]. Altogether, these data suggest that pro-inflammatory responses might be tightly controlled within the immunesuppressed testicular environment and only triggered by an early high level of viral replication (i.e. > 6 log_10_ PFU/mL, as observed for MuV at peak level on day 3) or upon the activation of specific pathogen recognition receptors, such as TLR2, as seen with Mumps [22]. Nevertheless, a notable limitation of our *ex vivo* model is its incapacity to recapitulate the impact of systemic infection and widespread inflammation on the testis. Inflammatory cytokines are known to disrupt the blood-testis barrier (BTB) (e.g. SARS-CoV-2 [51] and Mumps virus [52] in mice models), potentially facilitating the egress of infected cells and virions into the seminiferous tubule lumen and semen. In the one experimentally infected macaque testis analyzed, no CD45 + cell infiltrates were detected in testis at d2pi (not shown). However, we cannot draw any conclusion from this single animal and early time point. Since the role of systemic inflammation on the testis during CHIKV infection could not be addressed in our study, further investigations are required. The prolonged presence of CHIKV RNA in semen of infected men despite negative viremia suggests the viral RNA in semen is not a result of passive diffusion from the blood [16]. Whether the testis or other male genital organs are primary producing sources remains unclear. In one case report, the patient reported urethral burning 3 days prior symptoms onset, and CHIKV was concomitantly detected in both urine and semen, which could indicate an urethral infection [10]. The urethral mucosa is known to be rich in macrophages, which have been shown to act as reservoirs for HIV [53,54] and represent potential targets and reservoirs for CHIKV. This suggests a potential mechanism for localized viral persistence and shedding from the urethra. In a prospective cohort study, 6 out of 42 men had a CHIKV positive semen sample (with a detection up to 56 days in one patient) and 5 had positive urine samples [11]. CHIKV RNA was detected for over 30 days in all fluids, the longest shedding duration being in urine (95 days). Urine contamination could indicate infection of the kidney and/or urethra, the latter being also a potential source of viral RNA shedding in semen. Analyzing the nature of the infected cells within semen, as previously undertaken for ZIKV [12], will be crucial to pinpoint the local productive sources of CHIKV (urethra, prostate, seminal vesicles, epididymis, and/or testis). Ultimately, a critical next step involves determining the infectivity of CHIKV in semen to fully ascertain its potential for sexual transmission.

Importantly, our study reveals that CHIKV infection has a detrimental effect on testicular cells *ex vivo*, especially PMCs. PMCs are crucial for male reproductive health, forming the outer border of the seminiferous tubules. Their essential functions include contracting to transport immotile sperm to the efferent ducts and secreting extracellular matrix proteins and factors that regulate the local environment for spermatogonial stem cell (SSC) maintenance. While other viruses like ZIKV, SARS-CoV2, and MuV also infect PMCs in human testis explants [13,21,22], CHIKV showed a more significant impact on this cell type. Thus, we observed increased PMC death upon CHIKV infection and a downregulation of a broad range of genes, including α-SMA, which was affected at both transcriptional and proteomic levels. This damage was most noticeable at d6 to d9pi, following the peak of infected cells and viral production at d3pi. The loss of contractile markers, such as *MYH11* and α-SMA, is notable as similar alterations have been observed in infertile patients [55]. This raises interrogations about a potential impact of CHIKV infection on sperm parameters *in vivo*. Beyond contractile elements, our *ex vivo* model also revealed a diminished expression of several extracellular matrix molecules due to CHIKV. The local regulatory network created by PMCs at basement membrane, in which Sertoli cells also participate, is key for supporting spermatogenesis [56]. For instance, MMP2, whose expression was decreased upon CHIKV infection, is essential for the remodelling of the blood testis barrier basal lamina and the release of spermatozoa in the lumen [56]. In addition, the diminished expression of *CXCL12*, a cytokine involved in SSC maintenance might negatively impact stem cell competence [57,58]. Testicular resident macrophages may also be impacted by CHIKV infection, as the transcriptional expression of polarization and activation markers such as *CD163* and *CD86* was affected. Whether these acute damages observed *ex vivo* can occur *in vivo* and lead to durable alterations of the testis homeostasis is unknown. Given these uncertainties, it is crucial to assess the effects of CHIKV infection on semen parameters in longitudinal samples.

Our study has several limitations. Given the limited access to human tissues, our sample size is relatively small (n = 7), and the donor ages are heterogeneous (29–79 years). Nevertheless, the statistical significance of our results in human testis explants underscores their robustness. Since testis tissue was available from only one infected macaque, robust *in vivo* conclusions are precluded. Furthermore, the intravenous route of infection may not fully represent the physiological dissemination of CHIKV. Nonetheless, the broad tissue tropism of CHIKV documented in fatal human cases suggests its potential to disseminate widely and reach various organs [7]. The low passage strain used in our study belongs to the Indian Ocean Lineage, which has been responsible for many epidemics across the globe since 2005, including a 2018 outbreak in Thailand [23]. It is part of the East-CentralSouth African (ECSA) genotype, a group that also includes the 2024–2025 strains currently circulating on Réunion Island [59]. The IOL strain acquired an E1-A226 V amino acid substitution in the E1 envelope glycoprotein during the 2005–2006 outbreak that conferred enhance infection and dissemination in *Aedes albopictus* [60]. New secondary adaptive mutations in E2 evidenced since 2006 also conferred a heightened ability to propagate in the main mosquito vector *Aedes albopictus,* without affecting CHIKV fitness in human cell lines [61]. These mutations were also evidenced in the currently circulating strains, along with newly described mutations [59]. The impact on testis tropism of mutations recently acquired by CHIKV strains is unknown. Another limitation of our *ex vivo* model is its inability to replicate the systemic inflammatory response that occurs during a complete CHIKV infection. Consequently, this model cannot assess how this broader immune reaction affects the testis, nor the infection's potential negative impact on men reproductive health.

In summary, this study demonstrates that CHIKV efficiently replicates in the human testis *ex vivo* and induces cell apoptosis, with a major impact on peritubular myoid cells. This could potentially have deleterious consequences for the reproductive health of infected men, as peritubular myoid cells play key roles for spermatogenesis and sperm transport [38]. Although CHIKV infectious particle production by testis explants declined over time and seminiferous cells were spared from infection, the testis's role as a potential source of prolonged CHIKV excretion in semen, particularly in an *in vivo* inflammatory setting disrupting the blood-testis barrier, cannot be dismissed.

These data, in the context of active CHIKV circulation, point to the necessity of human cohort studies. Such studies are now crucial for determining the consequences of CHIKV infection on semen quality, as well as the frequency, duration, infectivity, and origin of CHIKV shedding in semen.

## Supplementary Material

Supplemental Material

FigS4.tiff

FigS2.tiff

FigS5.tiff

FigS3.tiff

FigS1.tiff
