# Supplementary figures and images for: Chikungunya virus replicates in the human testis ex vivo and impacts peritubular myoid cells functional markers
Source: Emerg Microbes Infect. 2025 Dec 5;14(1):2587984. doi: 10.1080/22221751.2025.2587984 (PMC12683761; doi:10.1080/22221751.2025.2587984)

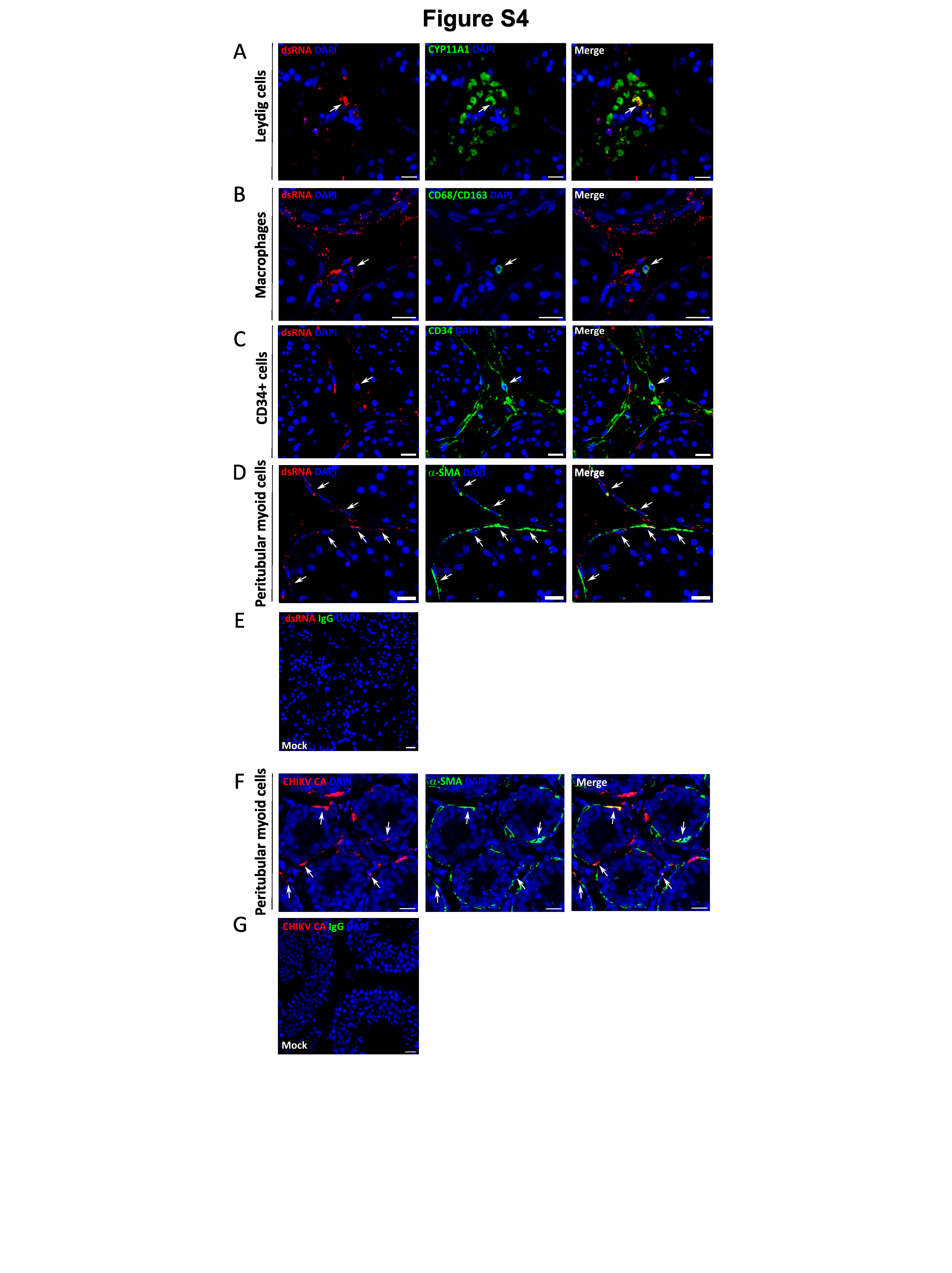

Supplement: FigS4.tiff [file TEMI_A_2587984_SM4533.tiff]

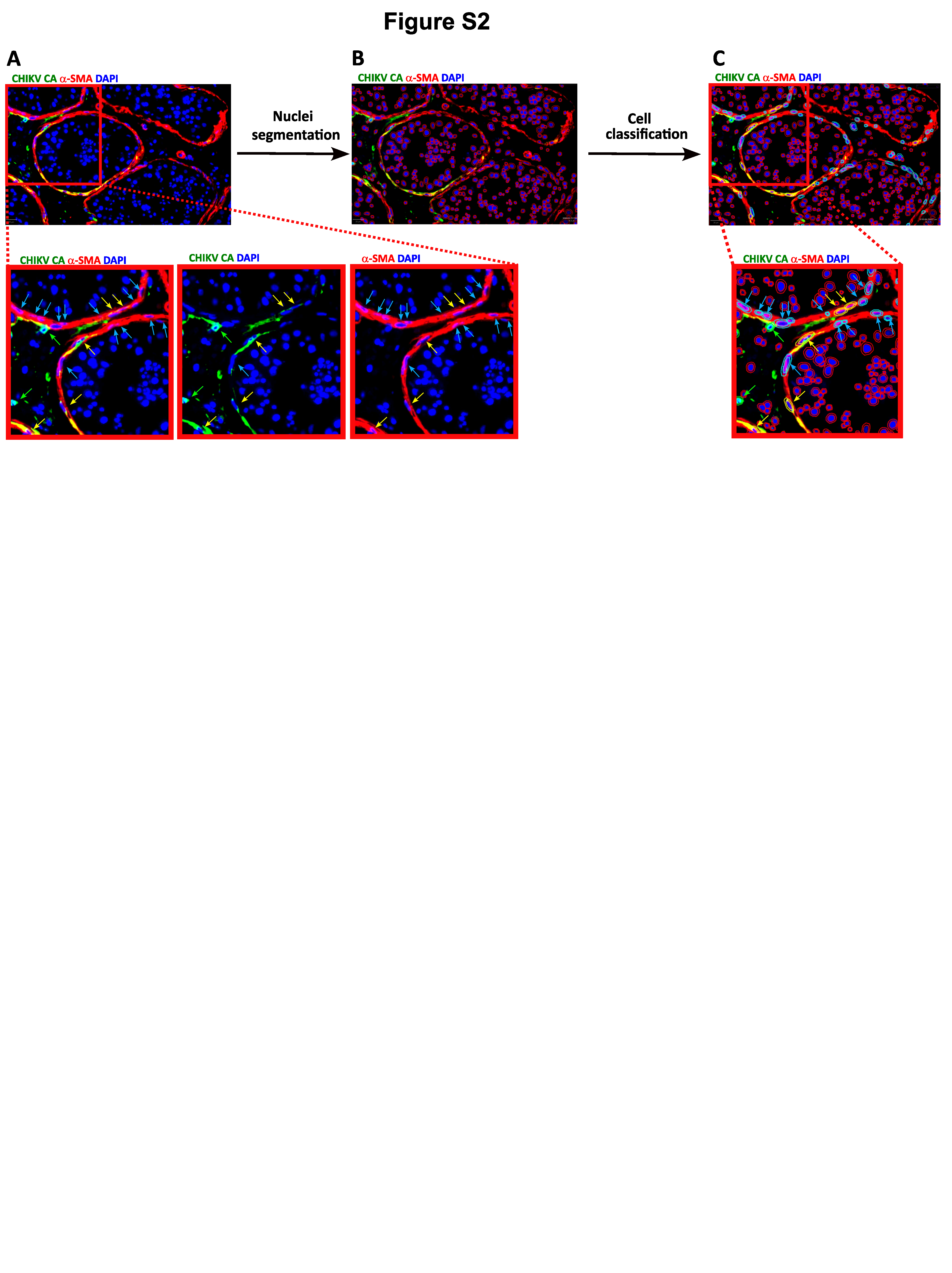

Supplement: FigS2.tiff [file TEMI_A_2587984_SM4532.tiff]

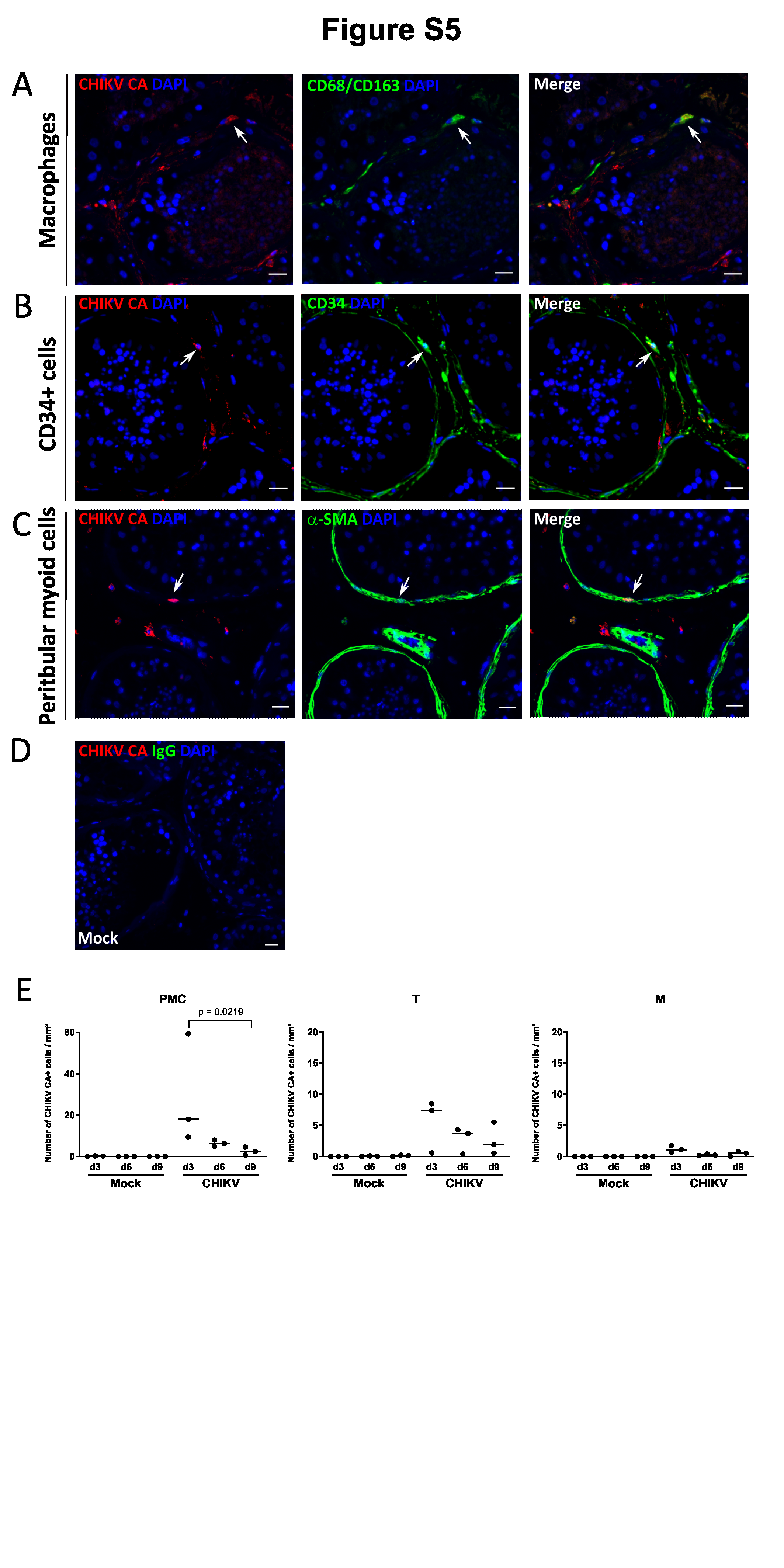

Supplement: FigS5.tiff [file TEMI_A_2587984_SM4531.tiff]

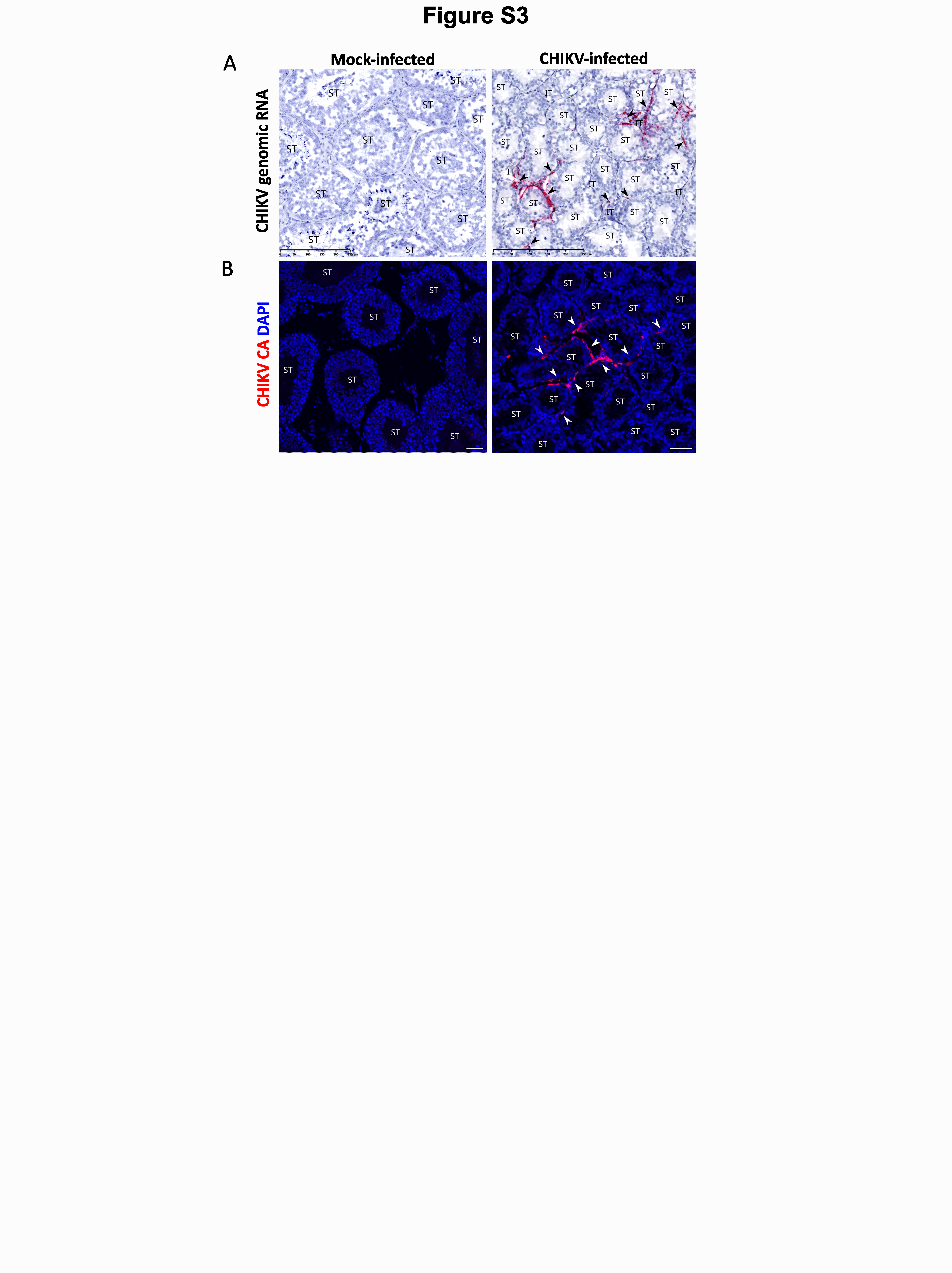

Supplement: FigS3.tiff [file TEMI_A_2587984_SM4530.tiff]

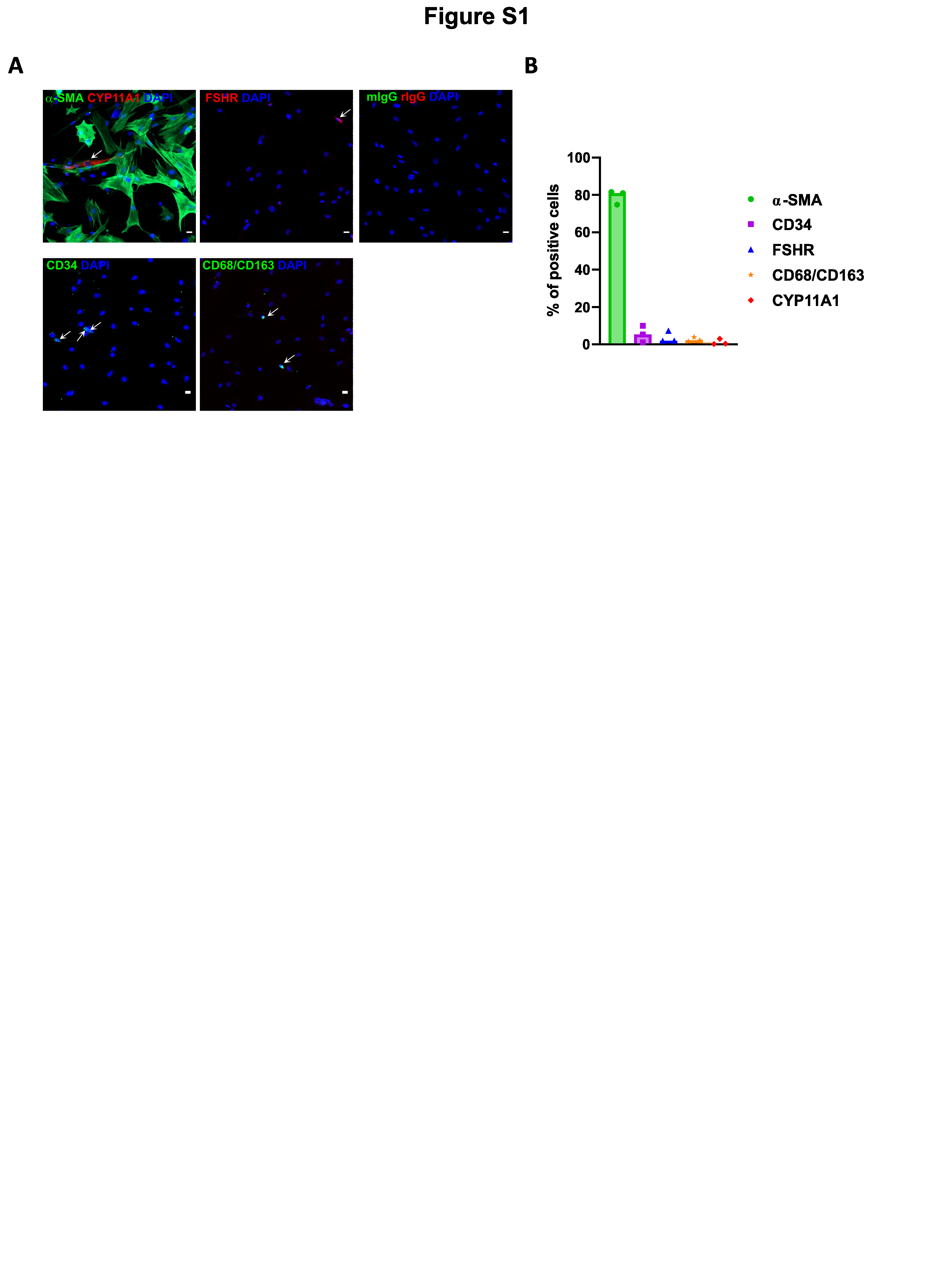

Supplement: FigS1.tiff [file TEMI_A_2587984_SM4529.tiff]
